# Melatonin Enhances the Anti-Tumor Effect of Fisetin by Inhibiting COX-2/iNOS and NF-κB/p300 Signaling Pathways

**DOI:** 10.1371/journal.pone.0099943

**Published:** 2014-07-07

**Authors:** Canhui Yi, Yong Zhang, Zhenlong Yu, Yao Xiao, Jingshu Wang, Huijuan Qiu, Wendan Yu, Ranran Tang, Yuhui Yuan, Wei Guo, Wuguo Deng

**Affiliations:** 1 Institute of Cancer Stem Cell, Dalian Medical University Cancer Center, Dalian, China; 2 Sun Yat-sen University Cancer Center, State Key Laboratory of Oncology in South China, Collaborative Innovation Center of Cancer Medicine, Guangzhou, China; 3 Department of Neurosurgery, Guangdong No. 2 Provincial People's Hospital, Guangzhou, China; 4 The First Affiliated Hospital, Dalian Medical University Cancer Center, Dalian, China; Georgia Regents University, United States of America

## Abstract

Melatonin is a hormone identified in plants and pineal glands of mammals and possesses diverse physiological functions. Fisetin is a bio-flavonoid widely found in plants and exerts antitumor activity in several types of human cancers. However, the combinational effect of melatonin and fisetin on antitumor activity, especially in melanoma treatment, remains unclear. Here, we tested the hypothesis that melatonin could enhance the antitumor activity of fisetin in melanoma cells and identified the underlying molecular mechanisms. The combinational treatment of melanoma cells with fisetin and melatonin significantly enhanced the inhibitions of cell viability, cell migration and clone formation, and the induction of apoptosis when compared with the treatment of fisetin alone. Moreover, such enhancement of antitumor effect by melatonin was found to be mediated through the modulation of the multiply signaling pathways in melanoma cells. The combinational treatment of fisetin with melatonin increased the cleavage of PARP proteins, triggered more release of cytochrome-c from the mitochondrial inter-membrane, enhanced the inhibition of COX-2 and iNOS expression, repressed the nuclear localization of p300 and NF-κB proteins, and abrogated the binding of NF-κB on COX-2 promoter. Thus, these results demonstrated that melatonin potentiated the anti-tumor effect of fisetin in melanoma cells by activating cytochrome-c-dependent apoptotic pathway and inhibiting COX-2/iNOS and NF-κB/p300 signaling pathways, and our study suggests the potential of such a combinational treatment of natural products in melanoma therapy.

## Introduction

Melanoma is one of the most aggressive forms of skin cancer, which has been occurring with an increased incidence faster than that of any other cancer in the world [1]–[3]. Although melanoma in early stage is curable, the prognosis and overall survival for patients with metastasized melanoma is unfavourable. Patients in metastasis even have a median survival of only 6–10 months [4]. Melanoma is characterized by the forming of resistance to cytotoxic agents during the progression, and the effective treatment options for it are very few. Therefore, discovering and confirming new cytotoxic agents exerting anti-melanoma activities becomes very important. More and more natural products extracted from plants and animals have been shown to contribute to decrease cancer risks, and some of them even have been applied in cancer chemoprevention and malignancy suppression due to their high efficiency, low toxicity and wide variety of sources [5]–[7].

Flavonoids has been suggested to be effective against cancer [8], [9]. Fistin is a major flavonoid extracted from many fruits and herbal sources, and found to exert anti-aging [10], anti-inflammatory [11], [12], and anti-viral [13]–[15] effects. Fisetin displays antitumor effects in many cancers, including inhibiting tumor cell growth, inducing tumor cell apoptosis, reducing tumor cell migration and invasiveness, inducing cell-cycle arrest in cancer cells, and so on [16]–[18]. However, its anti-cancer effectiveness is not powerful enough, and the use of high doses of fisetin is limited by the emergence of side effects. Therefore, the combinational treatments with other chemotherapeutic agents, especially natural antitumor compound, should be improved for fisetin, and the underlying mechanisms of such combination should also be identified to achieve higher potency.

Melatonin is a hormone widely found in animals, plants and microbes. It functions as a powerful antioxidant to protect nuclear and mitochondrial DNA from damage [19], [20]. In addition, as the main product of the pineal gland, melatonin has been attracting more and more attention by exerting anti-proliferative, pro-apoptotic, and anti-angiogenic properties in multiple types of cancer cells [21]. Although the underlying molecular mechanism of antitumor activity for melanoma has not been fully elucidated, various studies *in vivo* and *in vitro* demonstrated that it might be partially realized through inhibitions of MMP-9 and NF-κB [22], blocking HIF-1α, STAT3 signaling and VEGF expression [23], regulating the transcription of cell proliferation-related genes, such as Nestin, Bmi-1 and Sox2 [24], suppressing the expression of 45S pre-ribosomal RNA and upstream binding factor [25]. Based on its ability to affect multiple signaling pathways, its contribution to diverse physiological functions and its very few side-effects, it might potentially be a suitable candidate to serve as a partner of other chemopreventive or chemotherapeutic agents to form a better and novel treatment strategy for cancer. The underlying molecular mechanisms of such combination also deserve better investigation to achieve additional benefits in cancer therapy.

The aberrant or increased activity of COX-2 and the high levels of its product PGE2 are observed in a variety of cancer types, especially in colon cancer [26], [27]. Many reports have demonstrated the wide range of effects for COX-2 product PGE2 in carcinogenesis and tumor development, including inducing angiogenesis, promoting cellular proliferation, inhibiting cell apoptosis, stimulating tumor invasion, and so on [28]. In melanoma, COX-2 is associated with tumor progression [29]–[31].Similarly, inducible NO synthases (iNOS) and its product NO have also shown overexpression in different types of cancer, including melanoma. iNOS is overexpressed in most cultured melanoma cells and in human melanoma samples [32], [33]. Moreover, its expression has been found to be a strong predictor of disease-specific and overall survival (OS) for stage III melanoma patients [34]. Although numerous reports suggested the cytotoxity of overexpressed NO to cancer cells, the continuous low expression of intracellular NO like PGE2 has been shown to promote tumor progression and induce anti-apoptotic effects in many tumor types. Thus, COX-2 and iNOS represent prime targets for potential treatment of cancer. Some natural products extracted from plants have been studied to be able to selectively inhibit COX-2 and iNOS expression, and have potential to act as anti-cancer molecules [35]–[38]. Both COX-2 and iNOS expression are strictly controlled transcriptionally by the recruitment of transactivators and coactivators, such as NF-κB and p300, to their key promoter regions [39]–[41]. However, whether fisetin could regulate COX-2 and iNOS expression and whether melatonin could sensitize or enhance such regulation to further inhibit melanoma cell growth remains unclear.

In this study, we determined whether melatonin could potentiate or enhance fisetin's antitumor effect in human melanoma cells. We also obtained mechanistic insight into the action mode of such combination. The combinational effects of fisetin and melatonin on tumor cell proliferation, migration, and apoptosis in melanoma cells were analyzed. The expression changes of some key proteins involved in cell proliferation and apoptosis signaling pathways and the changes of their upstream regulatory factors were further analyzed to uncover the underlying molecular mechanisms of this combinational mode. The findings from this study showed the potential of such a combinational treatment in melanoma therapy.

## Materials and Methods

### Reagents

Fisetin, 1400W, celecoxib (CB) were purchased from Sigma-Aldrich (St. Louis, USA). Melatonin was purchased from J&K Chemical Ltd. All reagents were dissolved in DMSO as the initial concentrate and diluted with medium before adding to the cell.

### Cell lines and cell culture

The human melanoma cell lines MeWo and Sk-mel-28 were cultured as monolayer in RPMI 1640 media supplemented with 10% fetal bovine serum, 100 U/ml penicillin and 100 µg/ml streptomycin and grown under 5% CO_2_ at 37°C.

### MTT assay

Cell viability was measured by a MTT assay. Five replicated wells were used for each concentration.

### Cell migration assay

Cell migration was detected by a scratch assay. Briefly, melatoma cells were grown to full confluence and treated with different does of fisetin or melatonin. The confluent cell monolayer was scraped in a straight line to create ‘scratches’. The images of the wound gaps were photographed using a phase-contrast microscope.

### Clone formation assay

Melanoma cells were plated in 6-well plates overnight and then treated with fisetin or melatonin for 24 hrs. The cells were trypsinized into single cells and were seeded into a 6-well plate at 1000 cells/well. After being incubated at 37°C with 5% CO_2_ for 14 days, the cells were washed with PBS and fixed with the mixture (methanol∶glacial∶acetic 1∶1∶8) for 10 mins, and stained with 0.1% crystal violet for 30 mins. The clones with more than 50 cells were counted under an optical microscope.

### Apoptosis assay

Apoptosis was measured by Annexin V staining–based FACS analysis.

### Western blot analysis

Total cell lysates were separated in a 10% SDS-PAGE and electrophoretically transferred to a PVDF membrane. The proteins were blotted using antibodies and visualized by enhanced chemiluminescence. The antibodies against β-actin, GAPDH, PARP, iNOS and COX-2 were obtained from Cell Signaling Technology and the antibodies against p300, NF-κB p50 and p65 and cytochrome-c were purchased from Santa Cruz Biotechnology.

### Confocal immunofluorescence

The cells grown on chamber slides and fixed with 4% paraformaldehyde for 30 mins, permeabilized with 0.2% TritonX-100 for 5 mins, and blocked with 10% bovine serum albumin (BSA) in PBS for 30 mins. The antibodies against p300, p50 or p65 (Santa Cruz) were added to the cells and incubated overnight at 4°C. Non-immune IgG was included as controls. The fluorescein isothiocyanate- and rhodamine-conjugated secondary antibodies diluted in blocking solutions were added and incubated for 1 hr. The cells were detected with a Leica confocal microscope and images were processed with Image-Pro Plus 5.1 software.

### Pulldown assay

The nuclear proteins (400 µg) were mixed with double-strand biotinylated COX-2 promoter probe (4 µg), streptavidin agarose beads (50 µl) in 500 µl PBSI buffer containing 0.5 mM PMSF, 10 mM NaF, 25 mM β-glycerophosphate and rotated for overnight at RT. The beads were centrifuged, washed with PBSI buffer, resuspended with SDS-PAGE loading buffer and boiled at 95°C. The supernatant was analyzed by Western blot.

### Immunoprecipitation assay

The nuclear proteins were incubated with the antibodies for 3.5 h at 4°C. The protein-A/G agarose beads were added and the mixture was incubated at 4°C overnight. After washing with ice-cold PBSI buffer, the beads were mixed with loading buffer and boiled. The precipitated proteins were separated by SDS-PAGE and detected by Western blot analysis using the acetylated-antibodies.

### Statistics analysis

Every experiment was done three times, and the mean values and standard deviations were calculated using origin 8.0 software. P<0.05 was considered to be statistical significance.

## Results

### Melatonin enhanced fisetin-medited cell proliferation inhibition

**Figure 1:**
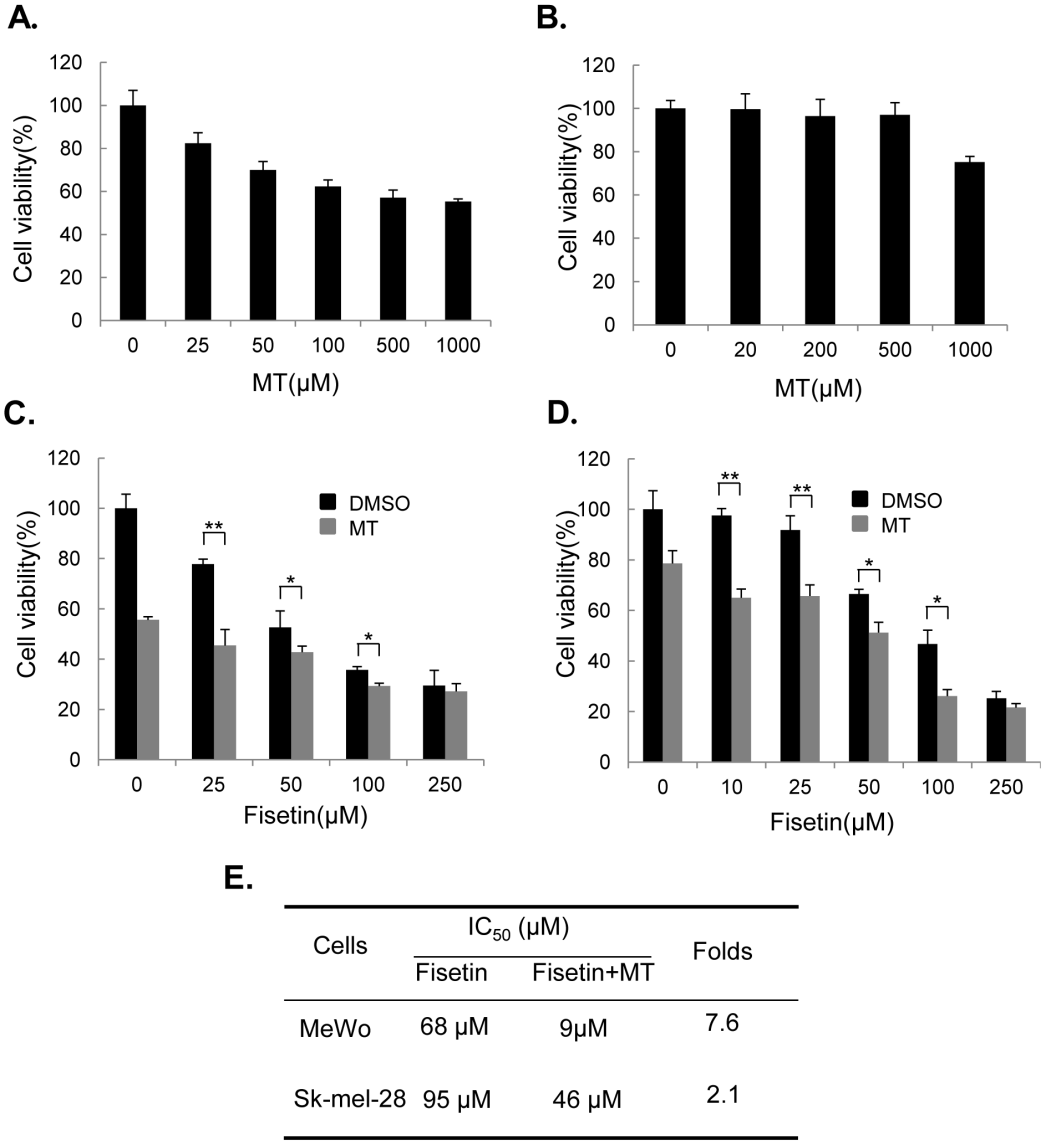
Melatonin enhanced fisetin-mediated inhibition of cell proliferation. (**A–B**), Human MeWo (**A**) and Sk-mel-28 (**B**) cells were treated with the indicated doses of melatonin (MT) for 48 hours, and the cell viability was examined by MTT assay. (**C–D**), MeWo (**C**) and Sk-mel-28 (**D**) cells were treated with the indicated doses of fisetin (F) alone or combined with MT (1 mM) for 48 hours, and the cell viability was examined by MTT assay. (**E**), The IC_50_ values of fisetin (F) for cell viability inhibition in cells treated with or without melatonin (MT) were determined. The data is presented as mean ± SD of three separate experiments. *, *P*<0.05, a significant difference compared to the control group.

MTT assay was used to test the combined effects of fisetin and melatonin on cell viability in MeWo and Sk-mel-28 cells. Melatonin alone at the dose from 20 µM to 1000 µM suppressed melanoma cell viability in a dose-dependent manner (Fig. 1A, 1B). However, the combinational treatment of the cells with melatonin (1.0 mM) with fisetin (0 µM–250 µM) significantly enhanced the inhibition of cell viability as compared with the cells treated with fisetin alone (Fig. 1C, 1D). The IC50 values of fisetin for cell proliferation inhibition in MeWo and Sk-mel-28 cells were next calculated. The combined treatment of fisetin with melatonin (1.0 mM) resulted in a marked reduction of the IC50 values of fisetin when compared to the cells treated with fisetin alone (Fig. 1E).

### Melatonin enhanced fisetin-mediated cell morphology change and clone formation inhibition

**Figure 2:**
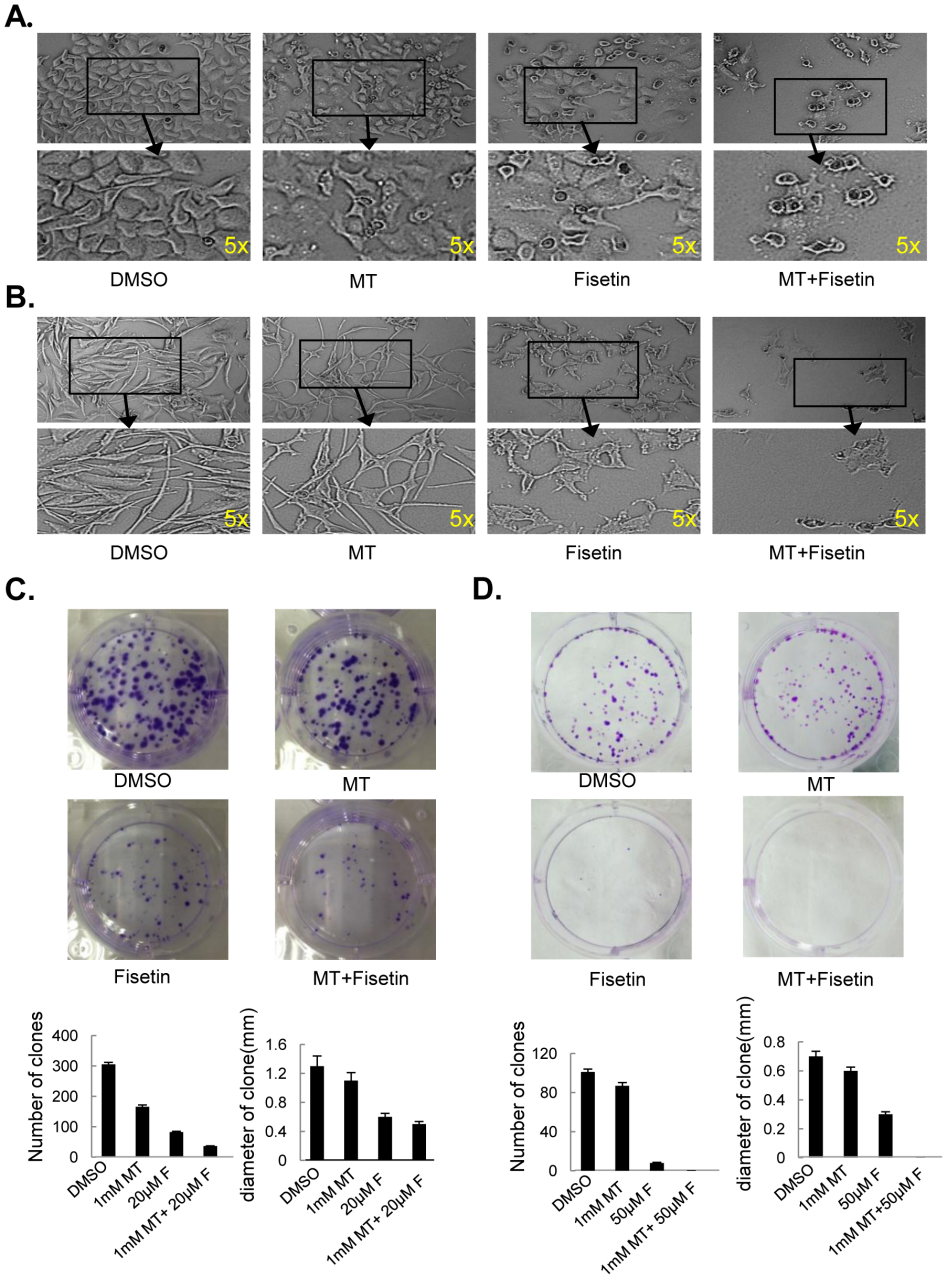
Melatonin enhanced fisetin-mediated cell morphology changes and clonal formation inhibitions. (**A–B**), The cell morphology changes of MeWo (**A**) and Sk-mel-28 (**B**) cells treated with fisetin (F) (20 µM) and melatonin (MT) (1.0 mM) for 48 h were observed. The cells were photographed using a microscope with a magnification of 40×10 fold. (**C–D**), Clone formation in MeWo (C) and Sk-mel-28 cells(D) treated with fisetin (F) (20 µM) and melatonin (MT) (1.0 mM) for 48 h were observed.

We next detected whether melatonin could sensitize fisetin-mediated changes in cell morphology and inhibition of clone formation in melanoma cells. The combined treatment led to a reduced cell-to-cell contact and more shrinkage and even broken cell morphology compared with the cells treated with melatonin or fisetin alone (Fig. 2A, 2B). Treated with melatonin or fisetin alone formed nearly 1000 clones after 7 days' growth, whereas combinational treatment of melatonin and fisetin considerably reduced clone formation, only 600 clones in MeWo cells (Fig. 2C) and 60 colones in Sk-mel-28 cells (Fig. 2D).

### Melatonin enhanced fisetin-mediated cell migration inhibition

**Figure 3:**
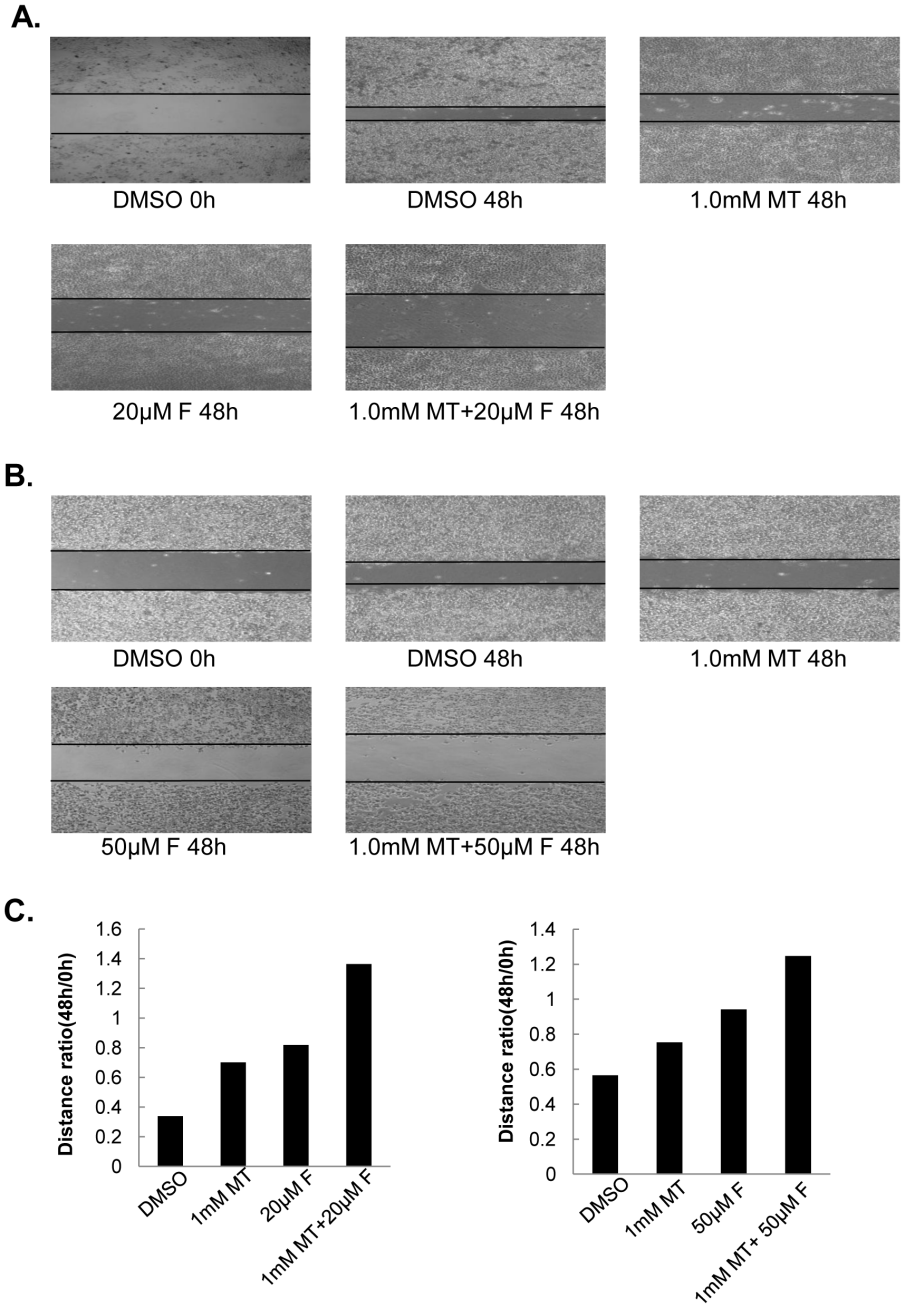
Melatonin enhanced fisetin-mediated cell migration inhibition. (**A–B**), MeWo (**A**) or Sk-mel-28 (**B**) cell monolayer grown to full confluence was wounded in a line and were left either untreated or treated with indicated doses of fisetin (F) or melatonin (MT) for 56 h. The images of the wound gap was observed with a magnification of 20×10 fold. (**C–D**), The quantitative analysis of the wound gap in MeWo (**C**) or Sk-mel-28 (**D**) cells treated with fisetin (F) or melatonin (MT) for 56 hours.

We employed a scratch assay to determine the combined effect of fisetin with melatonin on cell migration in melanoma cells. Treatment with melatonin or fisetin (20 µM) alone inhibited cell migration, but the combined treatment with melatonin markedly enhanced the fisetin-mediated inhibition of cell migration in MeWo cells (Fig. 3A). The wounding space between cell layers was occupied mostly by the migrating cells at 56 h, but the gap of the cells was not occupied by the migrating cells but a little enlarged in the MeWo cells cotreated with fisetin and melatonin (Fig. 3A). Similar enhancement of cell migration inhibition of fisetin by melatonin was also observed in melanoma Sk-mel-28 cells with combinational treatment of fisetin and melatonin (Fig. 3B).

### Melatonin increased fisetin-induced apoptosis

**Figure 4:**
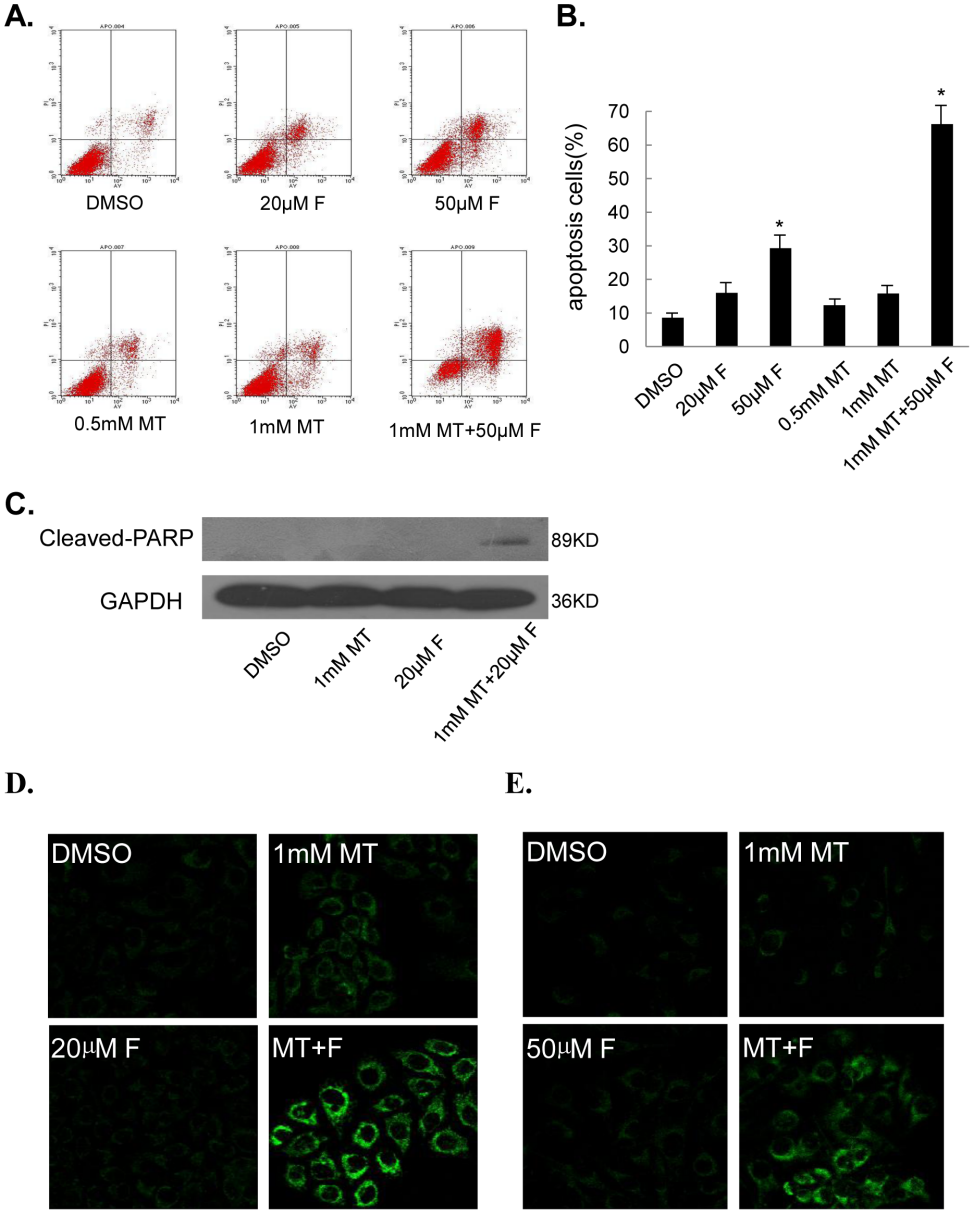
Melatonin enhanced fisetin–mediated apoptosis induction. Human MeWo cells were treated with 50 µM fisetin (F) and 1.0 mM melatonin (MT) for 48 hours. The apoptosis was determined by FACS analysis with annexin-V staining (**A, B**). The levels of the cleaved PARP protein (**C**) was analyzed by Western blot. The immunofluorescence imaging analysis was used to monitor the translocation of cyto-c in MeWo (**D**) and Sk-mel-28 (**E**). The data in (B) is present as the mean ± SD of three separate experiments. *, *P*<0.05, significant differences between treatment groups and DMSO control groups.

We analyzed the apoptosis in melanoma cells by FACS analysis. MeWo cells treated with fisetin alone at the concentrations of 20 µM and 50 µM respectively showed 16.5% and 29.6% apoptotic percentage at 48 hours after treatment (Fig. 4A, 4B). By contrast, the combination of fisetin with melatonin (1 mM) induced nearly 50% apoptotic cells in MeWo cells, resulting in an obviously increased pro-apoptotic effect (Fig. 4A, 4B). We then analyzed the expression changes of the cleaved PARP, one of the key proteins associated with apoptosis, in melanoma cells by Western blot. A marked induction of the cleaved PARP was deteced in MeWo cells cotreated with fisetin and melatonin compared with the cells treated with single agent (Fig. 4C). We also performed immunofluorescence (IF) analysis to monitor the subcellular localization of cytochrome-c (cyt-c), an upstream molecule of the caspase cascade-dependent apoptotic signaling pathway, in melanoma cells. Treatment with fisetin (20 µM) and melatonin (1 mM) alone for 48 h triggered the release of cyt-c from the inter-mitochondrial space into the cytosol in melanoma cells, but the combined treatment with two agents greatly improved the release of cyt-c (Fig. 4D,4E).

### Melatonin enhanced fisetin-mediated suppression of COX-2/iNOS signaling

**Figure 5:**
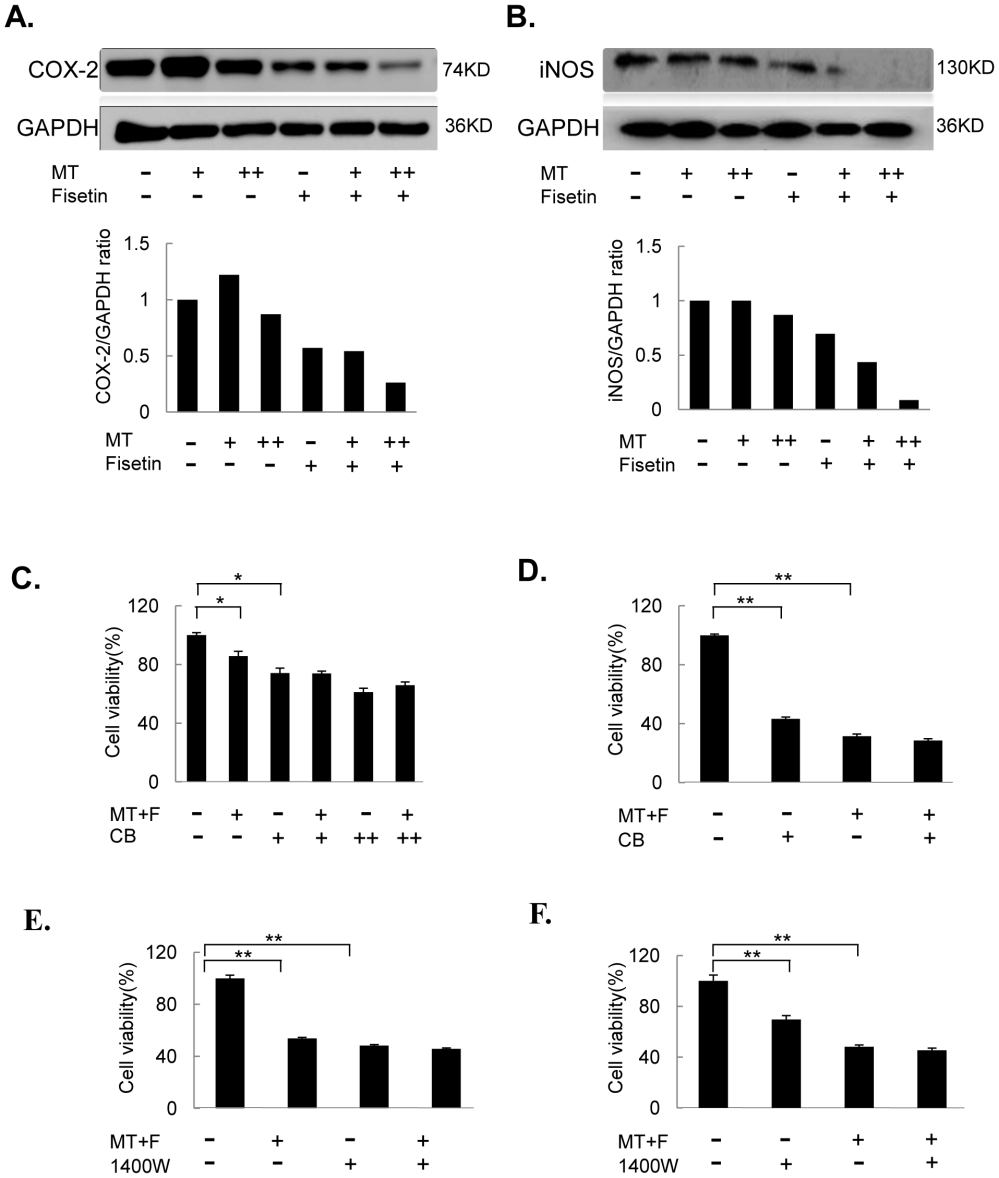
Melatonin enhanced fisetin-mediated suppression of COX-2/iNOS signaling. (**A–B**), The expression level of COX-2 protein (**A**) and iNOS protein (**B**) were analyzed by Western blot in human MeWo (**A**) or Sk-mel-28 (**B**) cells treated with the indicated doses of fisetin (F) (20 µM) and melatonin (MT) (1 µM or 1 mM) for 48 hours. “+” represents 1 µM MT, and “++” represents 1 mM MT. (**C–D**), MeWo (**C**) and Sk-mel-28 (**D**) cells were treated with F (20 µM) in combination with MT (1.0 mM) for 48 hours after pretreatment with the COX-2 selective inhibitor celecoxib (CB) (20 or 40 µM) for 24 hours, and the cell viability was determined by MTT analysis. For CB, “+” represents 20 µM, and “++” represents 40 µM. (**E–F**), MeWo (**E**) and Sk-mel-28 cells (**F**) were treated with the iNOS selective inhibitor 1400w (5 µM) for 24 hours, and then treated with F (20 µM) in combination with MT (1.0 mM) for 48 hours, and cell viability was determined by MTT analysis. Each data point was calculated from three triplicate groups and the data is presented as the mean ± SD. *, *P*<0.05, significant difference between treatment group and control group.

COX-2 and iNOS have been found to be involved in cell proliferation, migration and invasion in cancer cells. We detected the expression of COX-2 and iNOS at protein levels by Western blot. Treatment with fisetin alone at the dose of 20 µM or 50 µM slightly inhibited COX-2 or iNOS protein expression (Fig. 5A, 5B), while the combinational treatment with melatonin at the dose of 1.0 mM markedly reduced the expression of COX-2 or iNOS at protein level in melanoma cells (Fig. 5A, 5B). The MeWo and Sk-mel-28 cells were pretreated with COX-2-selective inhibitor celecoxib (CB) at a concentration of 20 µM for 8 h, and followed by the combined treatment with fisetin (20 µM) and melatonin (1 mM) for another 48 h. Treatment with CB alone inhibited cell proliferation, whereas CB pretreatment followed by a combinational treatment with fisetin and melatonin did not significantly alter cell viability inhibition mediated by the COX-2-selective inhibitor (Fig. 5C, 5D). Similarly, the co-treatment with fisetin and melatonin following pretreatment of iNOS-selective inhibitor 1400w in MeWo and Sk-mel-28 cells did not obviously elevate cell viability inhibition compared with fisetin and melatonin-induced anti-proliferation ability (Fig. 5E, 5F). These results indicate that the enhanced antitumor effect of the combinational treatment might partially be mediated through inactivating COX-2 and iNOS signalings.

### Melatonin enhanced fisetin-mediated inhibition of NF-κB/p300 signaling

**Figure 6:**
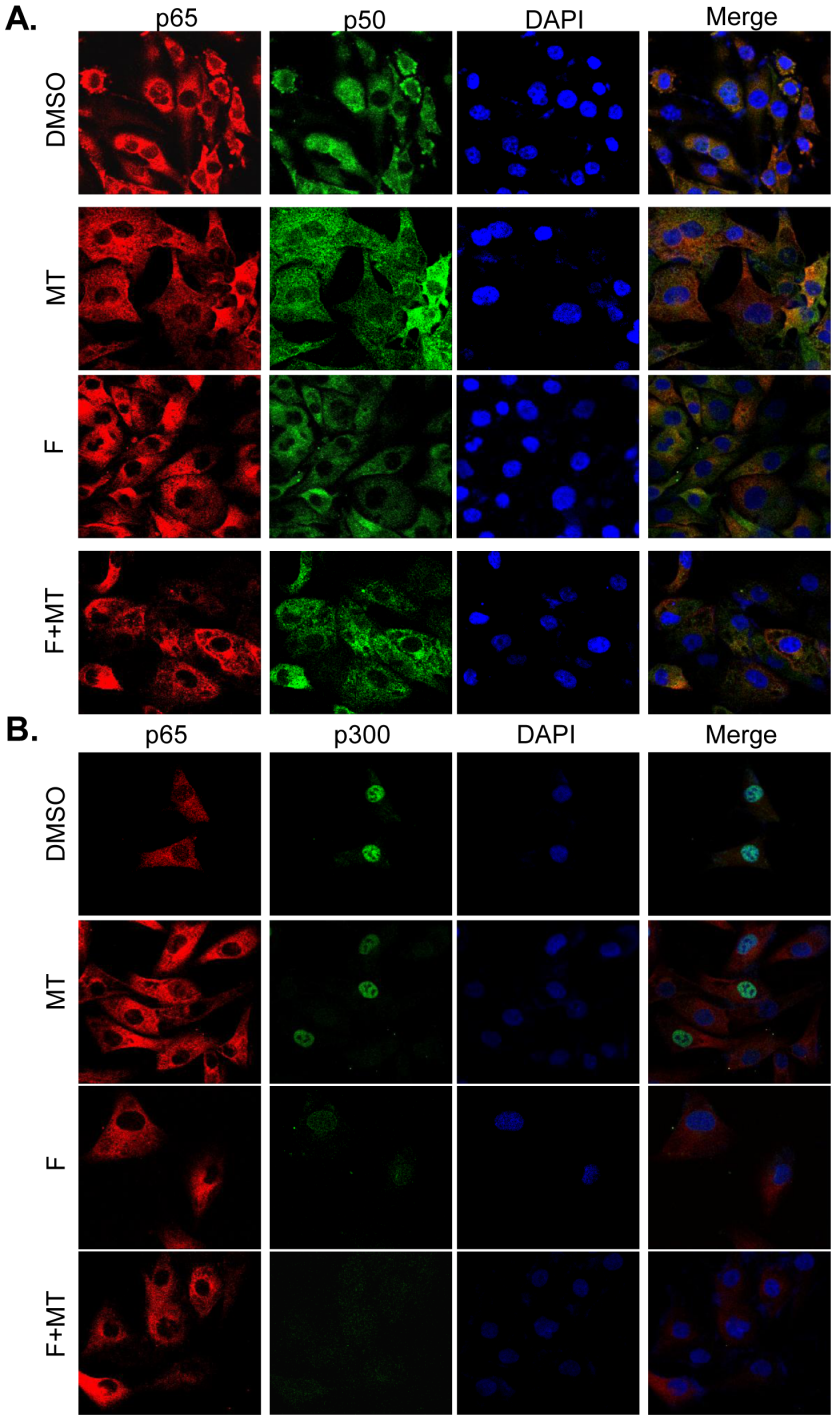
Melatonin enhanced fisetin-mediated nuclear translocation of p300 and NF-κB. The subcellular localization of p50, p65 and p300 and the co-localization of p65 with p50 (**A**) or p65 with p300 (**B**) in human MeWo cells treated with 20 µM fisetin (F) and 1.0 mM melatonin (MT) for 48 hours were examined by confocal microscopy. Cells with typical morphology were presented from more than 100 cells at each experiment.

**Figure 7:**
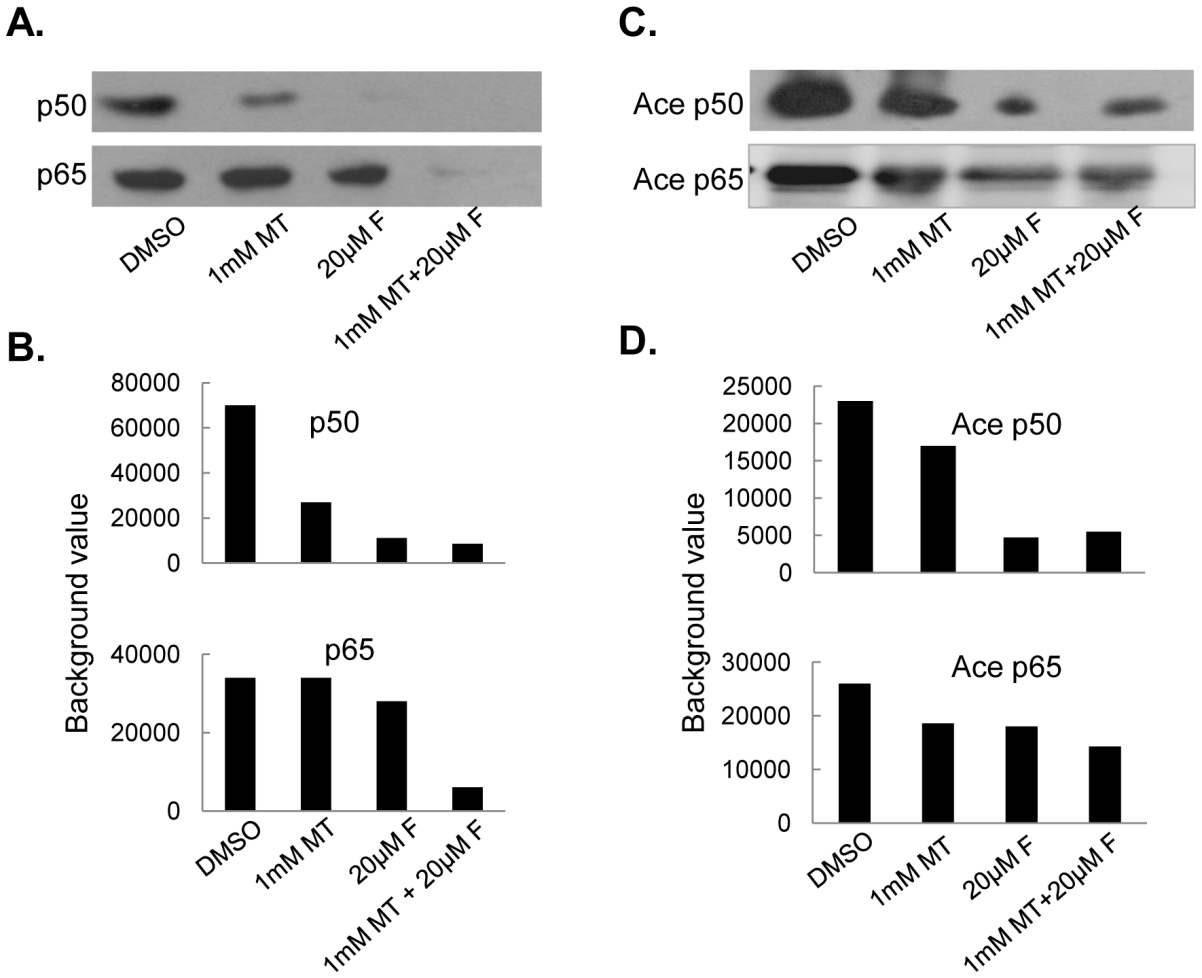
Melatonin enhanced fisetin-mediated inhibition of NF-κB binding and acetylation. (**A, B**), Human MeWo cells were treated with 20 µM fisetin (F) and 1.0 mM melatonin (MT). The binding of p50 and p65 to th biotinylated COX-2 promoter probe (**A**) was ananlyzed by pulldown and Western blot, the relative binding activities of p50 and p65 to COX-2 promoter (**B**) was analyzed by densitometric analysis. (**C, D**), MeWo cells were treated with F (20 µM) and MT (1.0 mM). Nuclear lysate from the treated cells was immunoprecipitated with antibodies against p50 and p65. The acetylated levels of p50 and p65 (**C**) was analyzed by Western blot, and the acetylated levels of p50 and p65 were quantitatively calculated by densitometric analysis (**D**).

COX-2 and iNOS expression are transcriptionally regulated by the binding of transactivator NF-κB to their promoter regions and the further recruitment of co-activator p300 interacting with NF-κB. We performed immunofluorescence assay to observe the localization and interaction of p300 and NF-κB in melanoma cells. The co-localization of p65 with p50 (Fig. 6A) or p300 (Fig. 6B) were detected in Sk-mel-28 cells. Treatment with fisetin alone induced the translocation of NF-κB p50/p65 (Fig.6A) and p300 (Fig. 6B) from the nuclei to cytoplasm, while the combined treatment with fisetin and melatonin enhanced the translocation of NF-κB p50/p65 (Fig. 6A) and p300 (Fig. 6B) from nuclei to cytoplasma. We next determined the binding activity of NF-κB on COX-2 promoter by a pulldown assay. As shown in Fig. 7A and 7B, the combined treatment with fisetin and melatonin markedly enhanced the inhibition of NF-κB p50 and p65 binding to COX-2 promoter compared to the treatment with fisetin or melatonin alone. As p300 has histone acetyltransferase (HAT) activity, we also determined the effect of the combinational treatment on p300-mediated acetylation of NF-κB p50/p65. We immunoprecipitated the nuclear extracted proteins from melanoma cell lines MeWo using anti-p50 or anti-p65 antibodies. The acetylated levels of these transactivators were tested using anti-acetyl-lysine antibody. The lower levels of the acetylated p50/p65 proteins were detected in MeWo cells co-treated with fisetin and melatonin than that in cell lines treated with single fisetin or melatonin alone (Fig. 7C, 7D).

## Discussion

The role of fisetin in killing cancer cells has been shown by its augmentative actions on the inhibition of cell proliferation and induction of cell apoptosis in different cancer cells. However, the anti-tumor effect of fisetin alone might not be powerful enough and adjunct therapy might be required to improve its efficacy in the treatment of cancer. Melatonin is a widely used antioxidant drug, and its anti-tumor activities have been proved by a number of studies [42]. Based on its multiple functions and low toxicity in cancer therapy, melatonin might be used in combination with other chemotherapeutic agents to improve therapeutic efficiency. In the present study, we have demonstrated that melatonin could potentiate fisetin-mediated antitumor effect in melanoma cells through activating cytochrome-c/caspase-dependent-apoptotic pathway, downregulating expression of COX-2 and iNOS through regulating the nuclear translocation of NF-κB and p300 and abrogating their binding on COX-2 promoter. To our knowledge, it might be the first time to report the effect of the combinational treatment of fisetin and melatonin on melanoma cells and to demonstrate the underlying mechanisms under such a combinational treatment.

The caspase-dependent apoptotic signaling is activated by the release of cytochrome-c from mitochondria and in turn activates the downstream execution starting with caspase-3 and the degradation of several essential cellular proteins such as PARP. The induction of apoptosis through activation of caspase cascade by fisetin in cancer cells, including colorectal carcinoma cells, HeLa cervical cancer cells, and prostate cancer cells, was reported in several previous studies [43]–[45]. Our present study also showed that the activation of caspase signaling cascade contributes to the enhancement of fisetin-mediated apoptosis by melatonin.

The COX-2 and iNOS signalings are implicated in the regulation of proliferation, apoptosis, migration and invasion responses in many kinds of cancer cells, and their inhibitors have been improved to be able to block survival signaling to accelerate killing tumor cells. The effective therapeutic strategy targeting COX-2/iNOS and their related key signaling molecules has been developed and is expected to provide important therapeutic implications in cancer treatment. Our present study detected the enhanced inhibition of COX-2 and iNOS expression in melanoma cells after co-treatment with fisetin and melatonin in comparison with single agent treatment, suggesting that inhibition of COX-2 and iNOS signaling at least partially contributed to melatonin potentiated fisetin-mediated cell proliferation inhibition in melanoma cells. Furthermore, we evaluated the possible molecular mechanisms of such suppression. Although the mechanism by which COX-2 and iNOS are up-regulated and over-activated in most cancer cells remains not clear enough, it was reported that their expressions were transcriptionally controlled by the binding of multiple transactivators such as c-Jun, C/EBPβ and NF-κB and coactivators such as p300 to the core promoter regions [46]. NF-κB shows a more extensive role and seems more essential for COX-2 and iNOS transcriptional activation. Similarly, p300 also plays a necessary role by exerting a global effect in maintaining COX-2 and iNOS promoter chromatin structure to increase recruitment of transactivators, such as NF-κB, and initiate COX-2 and iNOS transcription [47]–[48]. Our study confirmed the colocalization of NF-κB and p300 and the enhanced translocation of these two regulatory elements from nuclei to cytoplasm in melanoma cells after co-treatment with fisetin and melatonin through IF analysis. Moreover, we demonstrated that the increased inhibitory effects on COX-2 expression by the combined treatment with fisetin and melatonin was very possibly mediated by promoting p300 and NF-κB translocation from nuclear to cytosol and further inhibiting their binding to COX-2 promoter, thereby abrogating COX-2 transcriptional activation in melanoma cells. The same mechanism might apply to the elevated expression inhibition of iNOS signaling in melanoma cells treated by the combined fisetin and melatonin.

In conclusion, we found that melatonin enhanced the antitumor activity mediated by fisetin in melanoma cells. Furthermore, we elucidated the underlying molecular mechanisms of such enhanced action. The combined effects of fisetin and melatonin on melanoma cells might be achieved through activating cytochrome-c/caspase-dependent apoptotic signaling and inhibiting p300/NF-κB-mediated COX-2 and iNOS expression. These results not only indicate the great potential of natural products, such as fisetin and melatonin, in cancer chemoprevention and therapy, but also might serve as a basis and direction for the application of the combinational treatment of natural compounds in improving therapeutic efficiency for cancers.
